# Molecular Identification of *Trissolcus japonicus*, Parasitoid of the Brown Marmorated Stink Bug, by Species-Specific PCR

**DOI:** 10.3390/insects12050467

**Published:** 2021-05-18

**Authors:** Maple N. Chen, Ricardo D. Santander, Elijah J. Talamas, Peter J. Jentsch, Marie-Claude Bon, Srđan G. Aćimović

**Affiliations:** 1 Department of Biomedical Engineering, Cornell University, Ithaca, NY 14853, USA; 2 Hudson Valley Research Laboratory, School of Integrative Plant Sciences, Plant Pathology and Plant-Microbe Biology Section, Cornell University, Highland, NY 12528, USA; rd379@cornell.edu; 3 Department of Plant Industry, Florida Department of Agriculture and Consumer Services, Gainesville, FL 32399, USA; elijah.talamas@fdacs.gov; 4 Hudson Valley Research Laboratory, Department of Entomology, Cornell University, Highland, NY 12528, USA; pjj5@cornell.edu; 5 European Biological Control Laboratory, United States Department of Agriculture-ARS, 34980 Montferrier-sur-Lez, France; mcbon@ars-ebcl.org; 6 Alson H. Smith Jr. Agricultural Research and Extension Center, Department of Plant Pathology, Physiology, and Weed Science, Virginia Polytechnic Institute and State University, Winchester, VA 22602, USA; acimovic@vt.edu

**Keywords:** samurai wasp, PCR identification, species-specific primers, *Halyomorpha halys*, nondestructive DNA extraction

## Abstract

**Simple Summary:**

The brown marmorated stinkbug *Halyomorpha halys*, is an invasive pest that causes millions of dollars of crop damage each year in the US. A promising biocontrol agent for this pest is the samurai wasp, *Trissolcus* *japonicus*, which is a potential long term control method with few negative ecological impacts. However, the wasps’ small size of only 1 mm in length can make it difficult to identify morphologically. We developed a DNA-based approach to determine whether a wasp specimen is *T. japonicus* using conventional methods of DNA extraction, PCR, and gel electrophoresis. When tested against eight families of Hymenoptera, including three *Trissolcus* species, our method identified samurai wasp samples with 100% accuracy. Additionally, in silico analyses of various *T. japonicus* sequences provide evidence that the method will work throughout the US, Europe, and parts of Asia. We expect that this method will be a valuable tool for reliably identifying *T. japonicus* during distribution and recapture efforts to assess its survival, establishment, and dispersal for biocontrol purposes.

**Abstract:**

The samurai wasp, *Trissolcus japonicus* (Ashmead), has been proposed as a biocontrol agent against brown marmorated stink bugs (BMSB), due to its ability to parasitize and kill BMSB eggs. However, the wasps’ small size makes it challenging for those untrained in morphological identification to determine the wasps’ species. To circumvent this problem, a molecular method was created to identify *T. japonicus*. The method uses species-specific primers, designed in this study, which target the variable region of the mitochondrial Cytochrome Oxidase 1 (*CO1*) locus. After confirming successful DNA extraction from samples, the PCR amplification using our primers produced 227-bp PCR products for all *T. japonicus* specimens and no amplification in other microhymenoptera candidates. Additionally, DNA from BMSB-parasitized eggs gave positive PCR amplification, while the control BMSB samples showed no amplification. This indicates that PCR with our primers specifically and sensitively differentiates *T. japonicus* specimens from other similar wasp species and discriminates between *T. japonicus*-parasitized and non-parasitized BMSB eggs. Finally, an in silico analysis of *CO1* sequences demonstrated that our primers match the sequences of four different haplotypes of *T. japonicus*, indicating that our diagnostic method could potentially be applied to analyze *T. japonicus* populations throughout North America, Europe, and parts of Asia.

## 1. Introduction

The brown marmorated stink bug (BMSB), *Halyomorpha halys* (Stål), is an invasive pentatomid pest that is native to China, Japan, and other parts of East Asia. Beginning in the late 20th century, the BMSB spread from East Asia to the US in 1996 [1], Canada in 2010 [2], Switzerland in 2007 [3], and Italy in 2012 [4]. Wherever it spreads, BMSB damage crops by feeding on fruit including pears, peppers, and tomatoes, causing fruit wounds and fruit abscission [5,6]. In fruit trees, BMSB can cause damage to over 25% of a tree’s fruit. In 2010 alone, BMSB caused USD 37 million of crop damage in the mid-Atlantic US [5,7]. Looking towards the future, climate comparison models project that BMSB could spread throughout northern Europe, northeastern North America, southern Australia, New Zealand, and other locales between 30° and 50° in latitude [8].

A number of wasps have been observed to parasitize BMSB. Some are native to North America and Europe, including *Anastatus* sp., *Ooencyrtus* sp., *Telenomus* sp., and certain *Trissolcus* species. Of these, *Anastatus* has been shown to have the highest rates of parasitism in BMSB, especially *Anastatus reduvii* (Howard) in the US and *Anastatus bifasciatus* (Geoffrey) in Europe [9,10]. However, parasitoids native to North America typically show parasitism rates lower than 6% for BMSB, and often have difficulty developing inside and emerging from BMSB eggs [11]. In contrast, parasitoids native to East Asia including *Trissolcus japonicus* (Ashmead) and strains of *Trissolcus cultratus* (Mayr) consistently show higher parasitism rates, typically above 50%, and have greater rates of success for developing inside and emerging from BMSB eggs [9].

In particular, the samurai wasp *T. japonicus* has been considered as a promising biocontrol agent for BMSB in the US, since it can oviposit into and kill BMSB eggs at rates upwards of 70% [12]. In its native habitat of East Asia, a combination of chemical and biological control methods, together with *T. japonicus*, have reduced BMSB to an occasional agricultural pest [13]. In the United States, *T. japonicus* was first discovered in the wild in 2014 in Beltsville, MD, and has been found in the mid-Hudson Valley of New York State since 2016 [14,15]. *T. japonicus* shows preference for BMSB eggs over those of other pentatomids such as the spined soldier bug *Podisus maculiventris*, minimizing its off-target ecological impacts [12,16,17,18,19,20]. Compared to established BMSB control methods such as insecticides and nets, *T. japonicus* has the potential to be less costly [21,22,23,24,25,26,27,28], more target-specific, and it has demonstrated parasitism rates upwards of 80% [16]. *T. japonicus*’s ability to kill BMSB at the egg stage prior to pest feeding makes it a promising candidate for BMSB control. Combined with established integrated pest management techniques, crop damage due to BMSB could be controlled to occasional outbreak levels as it is in East Asia [13].

One challenge that researchers have faced is reliably and rapidly identifying the wasps during field release and recapture endeavors. These endeavors are essential for measuring the survival and establishment of the wasps. Current methods rely heavily on expert morphological identification, which is time consuming. Species of *Trissolcus*, including *T. japonicus*, are only about 1 mm in length, making identification difficult and labor-intensive for those without extensive training. Developing a simple method using standard molecular tools can provide reliable and fast results when identifying microhymenopteran candidates collected during survey. In this paper we designed, optimized, and validated a methodology for the PCR identification of *T. japonicus* individuals and *T. japonicus*-parasitized BMSB eggs using novel species-specific primers targeting the variable region of the mitochondrial cytochrome c oxidase subunit 1 (*CO1*) locus. The high mutation rate of the *CO1* locus allows it to be used to discriminate between closely related species and investigate intraspecific diversity and as such, is commonly used for genetic barcoding of species [29]. Species-specific primers were designed for use in monitoring survival of *T. japonicus* populations in the northeastern and mid-Atlantic United States. The method we present relies solely on species diagnostic PCR. Thus, we expect it to be a quick, accessible, and cost-efficient option to determine if a sample is *T. japonicus*, while still preserving a voucher specimen.

## 2. Materials and Methods

### 2.1. Primer Design

Candidate primers for specific identification of *T. japonicus* were designed based on 19 different partial *CO1* sequences at the 5′ end of the *CO1* locus. Criteria for selection of species used were that the species was either closely related to *T. japonicus* [30] or could be mistaken for *T. japonicus* by untrained technicians, especially in the mid-Atlantic US [31]. The species included *T. japonicus*, closely related Palearctic species *Trissolcus corai* Talamas, *Trissolcus kozlovi* Rjakovskij, *Trissolcus plautiae* (Watanabe), *T. cultratus*, and Nearctic species of the flavipes group including *Trissolcus euschisti* (Ashmead) [30]. The list also included more distantly related taxa that may be visually mistaken for *T. japonicus* such as *Telenomus podisi* Ashmead, Platygastridae sp., Cerephronidae sp., and Encyrtidae sp. (Table 1) [31]. These sequences were acquired from the NCBI Nucleotide Database. Factors considered when selecting the sequences included location, sequence completeness, and sequence length, and because this study was conducted on samples from the mid-Atlantic United States, *CO1* sequences from parasitoid wasps collected in North America were given preference whenever possible.

For primer design, 19 *CO1* sequences were first aligned using Codon Code Aligner (v.9.0.1) (https://www.codoncode.com/aligner/) (accessed on 19 March 2021). Primers were designed manually, taking advantage of single nucleotide polymorphisms between the aligned sequences. Regions of divergence between the species were AT-rich, so longer primers were selected to achieve an acceptable annealing temperature. In particular, 3′ mismatches between strands of G/A or C/C were favored, as they are shown to have the greatest inhibition ability compared to T/C, T/G, T/T, G/G, and C/A mismatches between strands [32,33,34].

To confirm that the candidate primers matched with target *T. japonicus CO1* sequences, 36 *T. japonicus* sequences from the US and Canada (MK188349, MW97094, MK188350, MW97071–MW97103) [35] were aligned with the primers using Unipro UGENE v38.1 [36]. Then, all available *T. japonicus CO1* sequences were obtained and aligned to our primers, regardless of location. All sequences used in this analysis were obtained from NCBI GenBank. Finally, potential off-target amplifications from the primers were conducted using Primer-BLAST [37]. *CO1* sequences from NCBI’s non-redundant nucleotide (nr) database from Hymenoptera (taxid: 7399) or Hemiptera (taxid: 7524) organisms were screened for possible amplification, with a minimum of 4 total mismatches with at least 2 mismatches at the 3′ end of the primers.

### 2.2. Sample Collection and Processing

Hymenopteran parasitoids were collected on 10 × 15 cm yellow sticky cards (Alpha Scents Inc., Canby, OR, USA) hung 1.5 m from the ground in trees and shrubs surrounding orchards throughout the mid-Hudson Valley and Finger Lakes region in New York. The cards were collected after 14 days, and a stereo microscope was used to identify wasp specimens. All samples collected in the field were morphologically identified to family, and sometimes to the genus and species level, ultimately representing eight families that could be mistaken visually for *T. japonicus.* Morphological identification was performed prior to removing the samples from the sticky cards and was done using taxonomic keys [38,39].

For the sample processing, small segments of the sticky cards containing parasitoids were carefully punched out from the yellow sticky card and debris was removed. The punch-out was submerged in synthetic mineral oil (Johnson & Johnson, New Brunswick, NJ, USA) in a 30 mL plastic cup for 8 h or until the sample could be gently removed from the card with a clean paintbrush. Then the insect was dried off on a cleaning wipe, (Kimberly-Clark, Irvine, CA, USA) transferred into a microcentrifuge tube containing 95% ethanol and incubated at room temperature for 2 h. Finally, the sample was rinsed with sterile distilled water before proceeding to the non-destructive DNA extraction. To avoid cross-contamination, the paintbrush used to collect and manipulate the insect specimens was dipped in a 10% bleach solution, then 70% ethanol, followed by distilled water between handling samples [40].

### 2.3. T. japonicus and BMSB Rearing Conditions

Some of the analyzed specimens came from colonies of *T. japonicus* and *H. halys* reared at the Hudson Valley Research Laboratory in Highland, NY. Laboratory-reared adult *T. japonicus* which were originally collected in Milton, NY, were maintained at 25 °C at 50% relative humidity and a 16:8 h light-dark cycle in a growth chamber and were fed diluted raw honey. Stink bug colonies, originally caught in Highland, NY, were maintained in rearing tents under similar conditions, and were fed organic green beans, carrots, sunflower seeds, habanero peppers, and jalapeno peppers. A pepper plant placed in the rearing tent was used for BMSB oviposition [41].

Laboratory-reared *T. japonicus* and *H. halys* samples were collected and placed at −20 °C to kill and store the specimens until use. Before DNA extraction, the specimens were thawed at room temperature.

### 2.4. Genomic DNA Extractions

Conventional genomic DNA extraction techniques involve destruction of the tissue sample, but for insect samples, it is desirable to retain an intact voucher for insect needle mounting and visual inspection. The insect voucher can be used to identify any samples for which PCR amplification was unsuccessful or to conduct morphological examination. For genomic DNA extraction, the DNeasy Blood and Tissue Kit (Qiagen, Hilden, Germany) was used, with modifications to make the process non-destructive to the insect voucher [42,43]. The intact sample was incubated in 180 μL Buffer ATL and 20 μL of 20 mg mL^−1^ proteinase K for 24–72 h at 55 °C, then the insect was removed and transferred to 70% ethanol for preservation. After adding Buffer AL to the lysate, the solution was incubated at 70 °C for 10 min, and the manufacturer’s protocol was followed. During elution, 50 μL of Buffer AE warmed to 55 °C was allowed to sit on the spin column membrane for 15 min before centrifugation to elute the DNA.

Amplifiability of the DNA extracted was assessed by PCR using universal primers for metazoan invertebrates LCO1490 (5′-GGT CAA CAA ATC ATA AAG ATA TTG G-3′) and HCO2198 (5′-TAA ACT TCA GGG TGA CCA AAA AAT CA-3′) [44]. This step serves to reduce the number of false negatives by identifying samples which had undergone inefficient DNA extraction or improper storage.

### 2.5. Polymerase Chain Reaction Conditions

Polymerase chain reaction (PCR) was performed in a 25 μL reaction volume including 0.625 activity units of DreamTaq DNA Polymerase (Thermo-Fisher Scientific, Waltham, MA, USA), 1X Green DreamTaq Buffer, 0.2 mM of each dNTP, 0.2 μM of each primer, and 3 μL template DNA per reaction. Conditions for thermal cycling included 4 min of initial denaturing at 95 °C, followed by 35 cycles consisting of 45 s of denaturing at 94 °C, 45 s of annealing at 58 °C, and 1 min of elongation at 72 °C, then a final elongation period of 5 min at 72 °C (Applied Biosystems 2720 GeneAmp Thermal Cycler, 96 wells, Thermo Fisher Scientific, Waltham, MA, USA). When verifying DNA extraction using the universal LCO1490/HCO2198 primers, an annealing temperature of 40 °C was used [43]. The optimal annealing temperature for the species-specific primers was determined experimentally using a gradient PCR ranging from 48 to 60 °C (Bio-Rad iCycler™ Thermal Cycler, 96 wells, Bio-Rad Laboratories, Hercules, CA, USA). Following PCR, 10 μL of the PCR product were run on a 1.5% agarose gel stained with 1X SYBR™ Safe DNA Gel Stain (Invitrogen, Carlsbad, CA, USA) together with 1 μL of 1 Kb Plus DNA Ladder (Invitrogen, Carlsbad, CA, USA), then visualized using a Gel Doc XR+ Imaging System (Bio-Rad Laboratories, Hercules, CA, USA).

### 2.6. Primer Specificity

An initial in silico analysis was conducted to assess the primers’ specificity against other common *Trissolcus* wasps that parasitize BMSB [30,45]. In this assay, we only selected species that we were not able to collect on sticky cards (Table 2).

Primers were then tested on genomic DNA extracted from morphologically identified specimens of *T. japonicus* to confirm successful amplification of a 227-bp band and correct sequence of the amplicon. Three randomly selected *T. japonicus* PCR amplicons were sequenced to verify that the amplified band matched the expected sequence. Then, to assess the specificity of the primers, 31 wasp specimens including *T. japonicus* and other morphologically similar and phylogenetically close species were tested with the species-specific primers after confirmation of successful DNA extraction (Table 3). These insect samples were collected with sticky cards, identified using taxonomic keys for insects [38,39], and their DNA extracted as described above. Sample selection was limited to insects collected in New York State, USA, which had been stored on yellow sticky cards at room temperature for fewer than three years.

### 2.7. Primer Sensitivity

In order to test the sensitivity of the novel primers designed in this study, the species-specific PCR reactions were run using different amounts of *T. japonicus* DNA in a 25 μL reaction: 50, 10, 5, 1, 0.5, 0.1, 0.05 and 0.01 ng. Due to the inability of NanoDrop spectrophotometer instruments to accurately report DNA concentrations lower than 5 ng μL^−1^, a concentrated DNA stock was extracted from pooling 30 *T. japonicus* wasps which was reliably detected using a NanoDrop 1000 UV/VIS spectrophotometer (Thermo Fisher Scientific, Waltham, MA, USA), then diluted to yield the appropriate DNA concentrations used in PCR.

### 2.8. Application to Parasitized Eggs

After *T. japonicus* emerges from parasitized BMSB eggs, the eggs are left largely empty. The ability to determine whether an empty egg has been parasitized by *T. japonicus* can be a valuable tool in determining the success of *T. japonicus* as a biological control agent, and molecular diagnostic assays have been successfully developed for other parasitoids [40,46].

To evaluate the ability of the primers to sensitively detect *T. japonicus* DNA in parasitized BMSB eggs, the primers were tested on eight samples consisting of empty (emerged) BMSB eggs previously parasitized by *T. japonicus*. To confirm the wasp species parasitizing the eggs, we collected samples from laboratory-reared *T. japonicus* colonies. After emergence of *T. japonicus* from BMSB eggs, eggs and wasps were placed at −20 °C for one week to kill the wasps. Then, the eggs were stored in a petri dish at room temperature until use. At the time of DNA extraction, eggs had been stored for two years under these conditions. Because of the lack of non-parasitized BMSB eggs when the assay was performed, we used three BMSB samples from lab-reared colonies to control for false positive PCR amplification of BMSB DNA.

In this assay, destructive DNA extraction using the DNeasy Blood and Tissue Kit (Qiagen, Hilden, Germany) was performed and confirmed using universal primers LCO1490/HCO2198, then the extracted DNA was amplified using the species-specific primers and run on an agarose gel.

## 3. Results

### 3.1. Species-Specific Primer Design and Optimization of PCR Conditions

Species-specific primer design was conducted using three *T. japonicus CO1* sequences from specimens collected in North America (MK188350, MK188360, MK188349), as well as *CO1* sequences of microhymenopteran species listed in Table 1. Two unique primers for *T. japonicus* were designed, TJ234F (5′-ATC CCA TCA TTA ATT TTA TTA ATC TAT AGG-3′) and TJ460R (5′-CAT GTA AAT AAC GTT CAA TTA TTA ATT GAT A-3′), which produced a theoretical 227-bp PCR amplicon (Supplementary Figure S1). When primers were tested in vitro using *T. japonicus* DNA as template, a PCR product of 227 bp was obtained. Sequencing and BLAST analyses of three randomly selected PCR products confirmed that the amplified DNA matched the *T. japonicus CO1* gene. No non-target amplification of *T. japonicus* DNA was observed at any of the annealing temperatures assayed, ranging between 48 °C and 60 °C. To reduce chances for non-specific amplification when analyzing DNA of other wasp species, we selected the highest annealing temperature of 58 °C that produced strong amplification of the target DNA.

**Figure 1:**
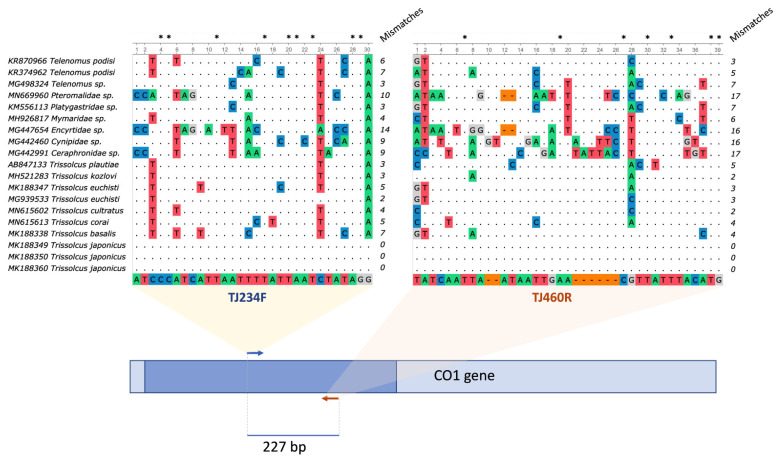
Alignment of TJ234F and TJ460R primers to various Hymenoptera *CO1* sequences, and location of primers within *CO1* locus. Detail of the *CO1* sequence alignment at the TJ234F/TJ460R primer binding sites of *T. japonicus* specimens used for primer design and other wasp species related to or coexisting in the same habitat as *T. japonicus*. Identical nucleotides in primers and the target sequences are marked with dots. Colored letters and dashes highlight nucleotide dissimilarities between primers and target sequences. The number of nucleotide mismatches is summarized in the column at the right side of each alignment. Asterisks above the alignments show the position of conserved nucleotides within primer sequences. The cartoon at the bottom of the figure represents an approximate location at the 5′ end of the analyzed partial *CO1* sequences (dark blue rectangle) and primer binding sites (arrows) within the complete *CO1* gene sequence (pale blue rectangle), as well as the theoretical amplicon size.

For the primer design, *T. japonicus CO1* sequences from US and Canadian specimens were aligned to sequences of other parasitoid wasps. The total number of primer mismatches between *T. japonicus* and the *CO1* sequences of the remaining wasp species ranged from five (*T. kozlovi*, MH521283; *T. euschisti*, MG939533) to 30 (*Encyrtidae* sp., MG447654). In most cases, mismatches accumulated in the 3′ regions of both primers (Figure 1). The Primer-BLAST [37] specificity analysis using the North American *T. japonicus* *CO1* sequences as templates and primers TJ234F/TJ460R confirmed a variable number of primer mismatches (≥4), including at least two mismatches at the 3′ end of both primers with relevant non-*T. japonicus* species.

### 3.2. Primer Specificity

The specificity of the TJ234F/TJ460R primer pair was tested in vitro with nine specimens of *T. japonicus* from three different locations within New York State, along with 22 other wasp specimens collected from locations where *T. japonicus* is present (Table 3). The samples consisted of parasitoids of BMSB including *T. euschisti*, *T. brochymenae*, and *Telenomus podisi*, which exhibit low rates of BMSB parasitism [16,30], as well as those that do not parasitize BMSB including samples of *Amitus* Haldeman (Platygastridae) and specimens in the families Pteromalidae, Myrmaridae, Encyrtidae, Eulophidae, Ceraphronidae, and Cynipidae [47,48,49,50,51,52,53]. Success of the non-destructive DNA extraction method used was confirmed, resulting in 81% of insect samples being accepted after PCR with the LCO1490/HCO2198 universal primers [44]. Using our species-specific primers, 100% of *T. japonicus* samples produced recognizable bands after PCR (*n* = 9), while none of the other 12 wasp species did (*n* = 22) (Table 3).

**Figure 2:**
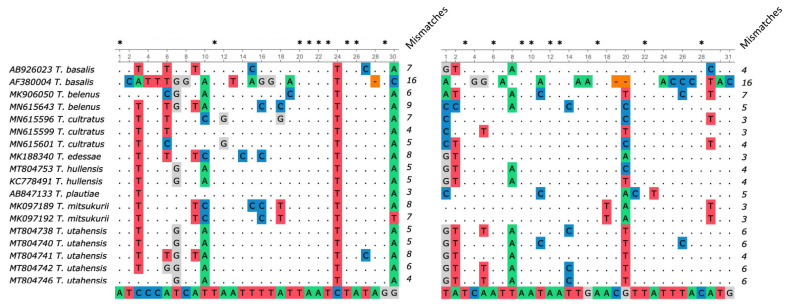
Alignment of TJ234F/TJ460R primers with sequences of eight BMSB parasitoids other than *T. japonicus*. Mismatch patterns of *T. basalis*, *T. belenus*, *T. cultratus*, *T. edessae*, *T. hullensis*, *T. plautiae*, *T. mitsukurii* and *T. utehensis* to our primers are shown. For each of the wasp species included in the figure, we analyzed a variety of sequences and identified diverse haplotypes. Frequently, two or more haplotypes showed the same pattern of mismatches across our two primer binding sites. Accordingly, a mismatch pattern in the figure may be found in one or more *CO1* haplotypes of the corresponding species. Dots denote identical nucleotides in primers and the target sequences. Colored letters or dashes indicate nucleotide dissimilarities, and the total number of mismatches for each species’ mismatch pattern is listed in columns to the right of the alignment. Asterisks indicate the position of conserved nucleotides within primer sequences. TJ234F/TJ460R are shown.

An in silico assay analyzed potential off-target annealing between our primers TJ234F/TJ460R and eight species of *Trissolcus* that parasitize BMSB, excluding *T. japonicus*. Alignment of all publicly available sequences for these species to our primers revealed that all eight species had mismatches to the species-specific primers (Figure 2). Numbers of mismatches between the *Trissolcus* sequences and the two primers ranged from seven (*T. cultratus)* to 32 (*T. basalis*), and all species showed a 3′ mismatch to the forward primer. All species except for *T. mitsukurii* showed 3′ mismatches to the reverse primer as well. Across the eight species analyzed, *T. basalis*, *T. belenus*, *T. cultratus*, *T. hullensis*, *T. mitsukurii*, and *T. utahensis* had two or more patterns of mismatch in the primer binding region, and each pattern is shown in Figure 2.

To test the applicability of our primers to larger populations of *T. japonicus*, a total of 103 *CO1* sequences were analyzed, representing populations from Europe (*n* = 11), North America (*n* = 36), and Asia (*n* = 56) (Table 4) [54,55]. Since the primers were intended for use in North America, these samples were analyzed first. Across the 36 *T. japonicus CO1* sequences collected from British Columbia (Canada) and 11 different states in the US, only one haplotype was observed, indicating that there is no apparent genetic variation in the barcoding region of the *CO1* locus within the United States and Canadian populations of *T. japonicus* [35]. The recorded partial *CO1* sequence of all North American samurai wasps matched the haplotype H2 reported by Stahl et al. [56], which was derived from a specimen collected in China (MH919759). This indicates that there is no evidence to suggest that the primers will not amplify *T. japonicus* DNA collected anywhere in the US.

After aligning additional *CO1* gene sequences from Europe and Asia to our primers, we found that the species-specific primers matched with 100% of the *CO1* sequences from Switzerland and Italy (11 sequences), 66.7% of the sequences from China (10 out of 15), 50% of the sequences from South Korea (1 out of 2) and 41% of those from Japan (16 out of 39). This represents a 71% of all *T. japonicus* sequences in NCBI GenBank.

Additional analysis based on the haplotype classification performed by Stahl et al. [56] revealed that primers are especially suited for the identification of *T. japonicus* haplotypes H1–H4 (Supplementary Figure S2), which include all the specimens from the US, Canada, Switzerland and Italy, plus variable percentages of specimens from China, South Korea and Japan (Table 4). The remaining haplotypes H5–H6, which have been reported only in Japan, showed a 3′ mismatch with the forward primer TJ234F, which could result in high false negative rates (Supplementary Figure S2) [56]. Finally, six haplotypes not previously described were categorized as haplotypes H7–H12. The six new haplotypes have only been reported in East Asia, and five of the six new haplotypes, H6 and H8-H12, showed at least one mismatch with our species-specific primers at the 3′ end, indicating that our primers are not well suited for identifying them (Table 4; Supplementary Figure S2). H7 showed only one mismatch to our primers, 6 bp from the 3′ end of the reverse primer TJ460R. In order to confirm the geographic locations of the haplotypes H1–H4 for which the species-specific primers will reliably amplify, all publicly available *T. japonicus CO1* sequences were categorized into twelve haplotypes, with their geographic origins noted (Table 4). The nucleotide markers used to distinguish between haplotypes at the *CO1* locus, including the six new haplotypes H7–H12 are shown in Supplementary Figure S4, and a barcode haplotype network linking haplotypes and geographic origins created with PopART [57] are provided as Supplementary Material (Supplementary Figures S3 and S4). Categorization of *T. japonicus* sequences confirmed that our species-specific primers TJ234F/TJ460R will reliably amplify samurai wasp specimens originating from North America and Europe, which are so far entirely composed of haplotypes H1–H4.

### 3.3. PCR Validation on Parasitized Eggs

Eight samples of BMSB eggs that had been parasitized by *T. japonicus* were analyzed along with three samples of BMSB DNA isolated from adult specimens’ legs. Out of the eight samples of parasitized and emerged BMSB eggs, all showed bands when amplified with the TJ234F/TJ460R primers, while none of the BMSB DNA samples showed positive amplification (Table 3). After destructive DNA extraction, no egg samples were rejected due to insufficient DNA, while one BMSB tissue sample was rejected.

### 3.4. Primer Sensitivity

**Figure 3:**
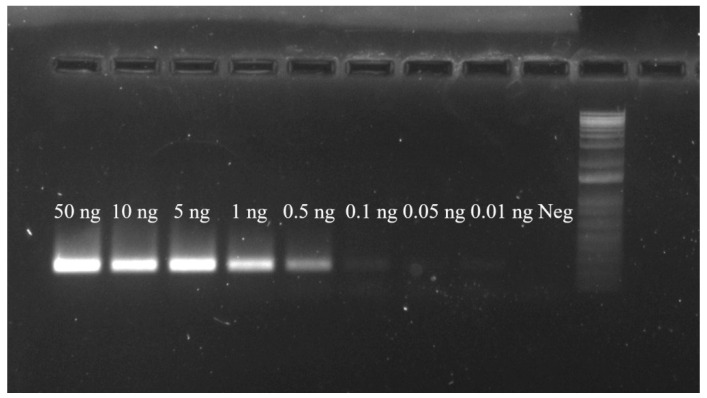
Sensitivity of the novel *T. japonicus* specific PCR using TJ234F and TJ460R primers. Total DNA amounts used in PCR were 50, 10, 5, 1, 0.5, 0.1, 0.05 and 0.01 ng, and a negative control (Neg) with 0 ng in a 25 μL reaction. The rightmost well contains 1 μL of 1 Kb Plus DNA Ladder (Invitrogen, Carlsbad, CA, USA).

After challenging the novel primers with *T. japonicus* DNA in amounts of 50, 10, 5, 1, 0.5, 0.1, 0.05 and 0.01 ng in a 25 μL PCR reaction, the primers produced the expected 227-bp band in samples receiving 0.1 ng or more of template DNA in a 25 μL reaction (Figure 3). Bright, recognizable bands were produced in samples containing greater than 0.5 ng of DNA in a 25 μL reaction volume, and the sample containing 0.1 ng of DNA showed a positive, faint band (Figure 3). Samples receiving 0.05 ng or less of template DNA in a 25 μL reaction did not produce bands bright enough to be reliably visualized under our current transillumination conditions.

## 4. Discussion

In vitro and in silico assays have demonstrated that our method reliably and sensitively detects *T. japonicus* DNA in both adult wasps and parasitized eggs. These results agreed with a previous Primer-BLAST (NCBI) analysis of the primers’ specificity, indicating that this method can be used reliably to distinguish samples of *T. japonicus* from those of tested wasps. For wasp species not tested in this study, such as *Trissolcus cosmopeplae*, additional study should be done to confirm the primers’ specificity. However, since this study analyzed the close relatives of *T. japonicus*, which have the most conserved *CO1* regions, the risk of non-target amplification of more distantly related *Trissolcus* species is low. The PCR using our primers is sensitive, producing bands at DNA concentrations as low as 0.1 ng in a 25 μL reaction and showing success for samples as old as 2 years since collection, stored on their yellow sticky cards at room temperature. During the control PCR step, 19% of samples were rejected due to low or degraded DNA, but more efficient DNA extraction techniques might be used to decrease this number. In future applications, the pairing of our single-step PCR method and a reliable DNA extraction method could lessen the need for a control PCR to samples that do not amplify with the species-specific primers. This alternative protocol has the potential to save time and money.

A broader in silico analysis of the target *T. japonicus* haplotypes H1–H4 [55] indicated that the primers show 100% homology with the target regions of the *CO1* locus for these haplotypes. These four haplotypes constitute 71% of the *T. japonicus* sequences in the NCBI database, including all the North American, Swiss, and Italian sequenced specimens, and 48% of the Asian sequenced specimens. In other haplotypes, including the six new haplotypes identified by examining publicly available *CO1* sequences, the primers showed at least one mismatch, including a 3′ mismatch with the forward primer TJ234F in seven out of eight remaining haplotypes (H6, H8–H12), that could result in false negative results. Haplotype H7 only showed one mismatch in reverse primer 6 bp from the 3′ end, suggesting that the TJ234F/TJ460R primers could potentially identify H7 haplotypes as well. However, further work will have to be done to validate the ability of our primers to anneal to H7 DNA. All eight haplotypes that display at least one mismatch (H5–H12) have only been reported in East Asia, indicating that the method will work in North America and Europe.

In tests on BMSB eggs parasitized by samurai wasps, 100% of parasitized eggs produced *T. japonicus*-specific diagnostic bands even in samples analyzed 2 years after the emergence of the wasps. Our species-specific primers produced no amplicons in control BMSB tissue samples. This expands the applicability of our PCR assay, as it can be used to determine if eggs were parasitized by *T. japonicus* without inspecting the emerged adults. For field studies, this could offer insight to the success of *T. japonicus* in parasitizing BMSB eggs.

Considering the agricultural importance of the samurai wasp, the ability to quickly identify *T. japonicus* specimens and *T. japonicus*-parasitized BMSB eggs is an important tool for present and future research. In the mid-Atlantic US, efforts are currently underway to redistribute the samurai wasp to mitigate crop damage from BMSB [31]. Surveying of redistributed *T. japonicus* is especially important to assess the ability of the adventive species to overwinter, the efficiency at which it parasitizes BMSB, the time frame in which parasitism tends to occur, and the identity of the parasitoid in the absence of the emerged insect itself [16,58]. In all these studies, a rapid identification method can facilitate the speed and accessibility of research.

Current methods require either morphological examination or PCR-amplification and sequencing of the variable *CO1* region [40,59]. In contrast, a PCR assay such as the one we present does not require the extra steps, time, or costs of sending samples for sequencing, or require the extensive training to accurately identify samples morphologically [60,61] The PCR designed in this study allows specific identification of *T. japonicus* using species-specific primers. Our method is faster than sequencing, which has a turnaround time of a few days, easy to interpret, and the materials required to perform a PCR are currently present in many laboratories. PCR and gel electrophoresis for molecular identification are established techniques for taxonomic identification of insect samples, but our method is unique because existing methods typically do not achieve species-level identification [40,46]. The ubiquity of PCR equipment allows many laboratories to conduct these analyses in-house, with shorter turnaround times than either of the prior methods, expediating studies on *T. japonicus* as a biocontrol agent for BMSB.

In sum, our method is both an accessible and reliable way to identify *T. japonicus*, a promising biocontrol agent for BMSB in the US. With redistribution efforts underway, the established range of the samurai wasp will likely increase, and so will demands for identification for the study of the parasitoid’s capacity to overwinter, parasitism rate, and potential effect on ecologically important pentatomids [15]. In this setting, the quick and inexpensive nature of our method will be a valuable tool for identification of the samurai wasp. Further work could aim to (i) create a multiplex PCR protocol combining both universal primers and primers specific for *T. japonicus*, dramatically reducing the workload of the method; (ii) optimize on our method for the *T. japonicus* haplotype and Hymenoptera biodiversity at different geographical locations; (iii) and/or develop multiplex or quantitative PCRs for simultaneous detection and identification of different *T. japonicus CO1* sequences or *Trissolcus* species.

## Figures and Tables

**Table 1:** *CO1* sequences from NCBI belonging to 15 different Hymenoptera species used for species-specific primer design.

Specimen ID	Family	Accession #
*Trissolcus japonicus* voucher TJ272	*Scelionidae*	MK188350
*Trissolcus japonicus* voucher TJ262	*Scelionidae*	MK188360
*Trissolcus japonicus* voucher Scel-0945	*Scelionidae*	MK188349
*Trissolcus euschisti* voucher Scel-0548	*Scelionidae*	MK188347
*Trissolcus euschisti* voucher FEMS015-09	*Scelionidae*	MG939533
*Telenomus podisi* isolate OH23	*Scelionidae*	KR870966
*Trissolcus plautiae* isolate: 121219-21	*Scelionidae*	AB847133
*Trissolcus basalis* voucher TSP228	*Scelionidae*	MK188338
*Trissolcus kozlovi* isolate T4	*Scelionidae*	MH521283
*Trissolcus corai* voucher USNM:ENT:01223976	*Scelionidae*	MN615613
*Trissolcus cultratus* voucher USNM:ENT:00977540	*Scelionidae*	MN615602
*Telenomus podisi* voucher BIOUG10919-B03	*Scelionidae*	KR374962
*Telenomus* sp. BIOUG24593-G02	*Scelionidae*	MG498324
Platygastridae sp. BOLD:AAZ4474 voucher BIOUG03482-G06	*Platygastridae*	KM556113
Mymaridae sp. INDOBIOSYS-CCDB25313-E11	*Mymaridae*	MH926817
Ceraphronidae sp. BIOUG32185-E11	*Ceraphronidae*	MG442991
Pteromalidae sp. BOLD:ADS5523 voucher CHARS00074-B06	*Pteromalidae*	MN669960
Encyrtidae sp. BIOUG32169-A04	*Encyrtidae*	MG447654
Cynipidae sp. BIOUG23888-C07	*Cynipidae*	MG442460

**Table 2:** *Trissolcus* wasps that parasitize BMSB and their GenBank accession numbers.

Species	*n*	Accession #
*Trissolcus basalis*	5	MK188338 AB926023–AB926024 MN615660 AF380004
*Trissolcus belenus*	8	MN615643–MN615644 MN603802–MN603806 MK906050
*Trissolcus cultratus*	8	MN615596–MN615603
*Trissolcus edessae*	2	MK188340 MN615575
*Trissolcus hullensis*	4	MT804753–MT804755 KC778491
*Trissolcus plautiae*	23	MN615614–MN615623 AB908183–AB908186 AB847147 AB847138–AB847142 AB847133–AB847135
*Trissolcus mitsukurii*	25	MK097189–MK097202 MN615586–MN615595 AB971831
*Trissolcus utehensis*	15	MT804738–MT804752

**Table 3:** Insect specimens evaluated by PCR in this study. The identification and origin of all insect samples for in vitro assays along with their collection year and PCR results.

*n* ^a^	Sample Type and Specimen Identification ^b^	Location	Collection Year	Control PCR ^c^	*Tj* Specific PCR ^d^
29	Field-collected insects				
	*Trissolcus japonicus*	Poughkeepsie, NY	2019	+	+
	*Trissolcus japonicus*	Poughkeepsie, NY	2019	+	+
	*Trissolcus japonicus*	Campbell Hall, NY	2018	+	+
	*Trissolcus japonicus*	Campbell Hall, NY	2018	+	+
	*Trissolcus japonicus*	Campbell Hall, NY	2018	+	+
	*Trissolcus japonicus*	Campbell Hall, NY	2018	+	+
	*Trissolcus japonicus*	Campbell Hall, NY	2018	+	+
	*Trissolcus euschisti*	Monroe County, NY	2018	+	−
	*Trissolcus euschisti*	Highland, NY	2020	+	−
	*Trissolcus brochymenae*	Huron, NY	2018	+	−
	*Trissolcus brochymenae*	Poughkeepsie, NY	2019	+	−
	*Telenomus podisi*	New Paltz, NY	2018	+	−
	*Telenomus podisi*	Milton, NY	2019	+	−
	*Telenomus* sp.	Campbell Hall, NY	2020	+	−
	*Telenomus* sp.	Poughkeepsie, NY	2018	+	−
	*Amitus* sp.	New Paltz, NY	2019	+	−
	*Amitus* sp.	Monroe County, NY	2019	+	−
	Pteromalidae sp.	Marlboro, NY	2019	+	−
	Pteromalidae sp.	Warwick, NY	2020	+	−
	Pteromalidae sp. or Myrmaridae sp. ^e^	Walden, NY	2019	+	−
	Encyrtidae sp.	Monroe County, NY	2019	+	−
	Encyrtidae sp.	Campbell Hall, NY	2018	+	−
	Eulophidae sp.	Monroe County, NY	2019	+	−
	Eulophidae sp.	Monroe County, NY	2019	+	−
	Eulophidae sp.	Huron, NY	2018	+	−
	Ceraphronidae sp.	New Paltz, NY	2018	+	−
	Ceraphronidae sp.	Holley, NY	2018	+	−
	Cynipidae sp.	New Paltz, NY	2019	+	−
	Cynipidae sp.	Williamson, NY	2019	+	−
13	Laboratory-reared specimens				
	*Trissolcus japonicus*	HVRL	2020	+	+
2	*Trissolcus japonicus*	HVRL	2020	+	+
8	*Halyomorpha halys* parasitized eggs ^f^	HVRL	2020	+	+
3	*Halyomorpha halys* ^g^	HVRL	2020	−	−

^a^*n*, Number of analyzed samples. ^b^ Field samples were collected using yellow sticky cards and identified based on morphology and other characters [38,39]. Laboratory-reared insects came from colonies maintained in the rearing facility at the Hudson Valley Research Laboratory (HVRL) in Highland, NY. ^c^ DNA extraction control PCR using the universal primers LCO1490/HCO2198 [44]. This assay was used to discard potential false negative reactions due to poor DNA extractions. ^d^ *Tj*, *T. japonicus* specific PCR designed in this study, using primers TJ234F/TJ460R. ^e^ Ambiguous specimen taxonomic identification based on morphological characters. ^f^ For the analysis we used parasitized and emerged *H. halys* eggs. DNA was extracted individually from 8 eggs. ^g^ DNA was extracted from legs of 3 adult specimens.

**Table 4:** Geographic origin and *CO1* haplotypes of *T. japonicus* analyzed in this study. Sequences of *T. japonicus* are grouped based on geographic origin down to the country or state, and haplotype(s) including those described by Stahl et al. (2019) [55].

Geographic Origin	Haplotype ^a^	Representative Sequences ^b^	*n* ^c^	Geographic Origin	Haplotype ^a^	Representative Sequences ^b^	*n* ^c^
Europe			11	Asia			56
Switzerland	H1	MH919753–MH919758		China			
Italy	H1 *, H3 *, H4 *	MK097184–MK097188			H2	MH919759	
North America			36		H2 *	MK188348	
Canada	H2 *	MK188349				MK188354–MK188355	
		MW97094				MK188358–MK188359	
US	H2 *					MK188361–MK188362	
California		MW97096				MN615624–MN615625	
District of Columbia		MW97073			H7 *	MK188357	
Delaware		MW97079			H11 ^#^	MK188363	
Maryland		MW97074				MN615630	
		MW97076			H12 ^#^	MK188353	
		MW97078				MN615632	
		MW97093		Japan			
New Jersey		MW97085–MW97086			H1	MH919743	
		MW97089			H1 *, H3 *, H4 *	MK188351	
		MW97091				MK188356	
New York		MW97082				MN615628	
		MK188350			H3	MH919744–MH919752	
		MW97084			H4	AB847131–AB847132	
Ohio		MW97097–MW97101				AB847136	
		MW97103			H5	AB908179–AB908182	
Oregon		MW97077				AB847144–AB847145	
		MW97087				AB894834–AB894835	
		MK188360				AB894838–AB894839	
Pennsylvania		MW97092			H5 *	MN615626	
Utah		MW97102			H6	AB847129–AB847130	
Virginia		MW97071–MW97072				AB847137	
		MW97088				AB847143	
Washington		MW97075				AB847146	
		MW97083				AB894836–AB894837	
		MW97090				AB894840–AB894841	
		MW97095			H8 ^#^	MN615627	
West Virginia		MW97080–MW97081			H9 ^#^	MN615633	
					H10 ^#^	MN615631	
				South Korea			
					H2 *	MK188352	
					H7 ^#^	MN615629	

^a^ Haplotypes H1–H6 were assigned to the reference sequences used by Stahl et al. (2019) [55]. Due to difference in sequence lengths, alignments with other *T. japonicus CO1* sequences involved shortening the compared sequences from 423 to 373 nucleotides. ^b^ NCBI accession numbers. ^c^ Number of sequences in the NCBI database from the indicated continent. * Denotes sequences that potentially match the haplotypes defined by Stahl et al. [55] Multiple haplotypes associated with a sequence (or group of sequences) indicates sequences potentially belonging to one of the indicated haplotypes. # Denotes sequences with haplotypes different from those described by Stahl et al. [55] based on the alignment of 373 nucleotide sequences, which have been arbitrary named H7–H12.

## Data Availability

All analyzed data is publicly available at NCBI Nucleotide Database: www.ncbi.nlm.nih.gov/genbank/ (accessed on 18 May 2021).

## Supplementary Materials

The following are available online at https://www.mdpi.com/article/10.3390/insects12050467/s1, Figure S1: Alignment of TJ234F/TJ460R primers against three *T. japonicus* sequences, Figure S2: Alignment between *T. japonicus* CO1 haplotypes and TJ234F/TJ460R primer mismatches, Figure S3: Barcode haplotype network of the *T. japonicus* CO1 sequences analyzed in this study, Figure S4: Position of haplotype markers in *T. japonicus* CO1 sequence.
