# Supplementary material for: Molecular Identification of Trissolcus japonicus, Parasitoid of the Brown Marmorated Stink Bug, by Species-Specific PCR
Source: Insects. 2021 May 18;12(5):467. doi: 10.3390/insects12050467 (PMC8157830; doi:10.3390/insects12050467)
Supplement: Supplementary file 1 [file insects-12-00467-s001.zip › insects-1187110-supplementary.pdf]

Supplementary Material

```
MK188349 Trissolcus japonicus      TAATTGGATCAGCAATAAGAATATTAATCCGAATAGAACTAAGAGTTCCAGGTATATTAATTGGTAATGA 70
MK188350 Trissolcus japonicus      TAATTGGATCAGCAATAAGAATATTAATCCGAATAGAACTAAGAGTTCCAGGTATATTAATTGGTAATGA 70
MK188360 Trissolcus japonicus      TAATTGGATCAGCAATAAGAATATTAATCCGAATAGAACTAAGAGTTCCAGGTATATTAATTGGTAATGA 70
                                   **********************************************************************

MK188349 Trissolcus japonicus      CCAAATTTATAATTCTATTGTAACTTCACATGCATTTATTATAATCTTTTTTATAGTTATACCAATTATA 140
MK188350 Trissolcus japonicus      CCAAATTTATAATTCTATTGTAACTTCACATGCATTTATTATAATCTTTTTTATAGTTATACCAATTATA 140
MK188360 Trissolcus japonicus      CCAAATTTATAATTCTATTGTAACTTCACATGCATTTATTATAATCTTTTTTATAGTTATACCAATTATA 140
                                   **********************************************************************

MK188349 Trissolcus japonicus      CTTGGAGGATTTGGAAATTGATTAATTCCTTTAATAATTAATGCCCCAGATATAGCATTTCCACGATTAA 210
MK188350 Trissolcus japonicus      CTTGGAGGATTTGGAAATTGATTAATTCCTTTAATAATTAATGCCCCAGATATAGCATTTCCACGATTAA 210
MK188360 Trissolcus japonicus      CTTGGAGGATTTGGAAATTGATTAATTCCTTTAATAATTAATGCCCCAGATATAGCATTTCCACGATTAA 210
                                   **********************************************************************

                                                                     TJ234F
MK188349 Trissolcus japonicus      ATAATATAAGATTCTGATTACTAATCCCATCATTAATTTTATTAATCTATAGGAATATCTTTGGATCAGG 280
MK188350 Trissolcus japonicus      ATAATATAAGATTCTGATTACTAATCCCATCATTAATTTTATTAATCTATAGGAATATCTTTGGATCAGG 280
MK188360 Trissolcus japonicus      ATAATATAAGATTCTGATTACTAATCCCATCATTAATTTTATTAATCTATAGGAATATCTTTGGATCAGG 280
                                   **********************************************************************

MK188349 Trissolcus japonicus      AACTGGAACTGGATGAACAGTATACCCTCCTTTATCTACTCAATTAAATCCCTCTATTGATTTAACTATT 350
MK188350 Trissolcus japonicus      AACTGGAACTGGATGAACAGTATACCCTCCTTTATCTACTCAATTAAATCCCTCTATTGATTTAACTATT 350
MK188360 Trissolcus japonicus      AACTGGAACTGGATGAACAGTATACCCTCCTTTATCTACTCAATTAAATCCCTCTATTGATTTAACTATT 350
                                   **********************************************************************

MK188349 Trissolcus japonicus      TTTTCCCTACATATAGCAGGAATTTCATCTATTCTTAGATCAATCAATTTCTTATGTACAATTATTAATA 420
MK188350 Trissolcus japonicus      TTTTCCCTACATATAGCAGGAATTTCATCTATTCTTAGATCAATCAATTTCTTATGTACAATTATTAATA 420
MK188360 Trissolcus japonicus      TTTTCCCTACATATAGCAGGAATTTCATCTATTCTTAGATCAATCAATTTCTTATGTACAATTATTAATA 420
                                   **********************************************************************

MK188349 Trissolcus japonicus      TAAGAAATATATCAATTAATAATTGAACGTTATTTACATGATCAATTTTAATTACAACAATTTTATTACT 490
MK188350 Trissolcus japonicus      TAAGAAATATATCAATTAATAATTGAACGTTATTTACATGATCAATTTTAATTACAACAATTTTATTACT 490
MK188360 Trissolcus japonicus      TAAGAAATATATCAATTAATAATTGAACGTTATTTACATGATCAATTTTAATTACAACAATTTTATTACT 490
                                   **********************************************************************
                                                      TJ460R

MK188349 Trissolcus japonicus      TCTATCTTTACCAGTTTTAGCAGGGGCAATCACTATAATTTTATCAGATCGAAATCTTAATACATCCTTT 560
MK188350 Trissolcus japonicus      TCTATCTTTACCAGTTTTAGCAGGGGCAATCACTATAATTTTATCAGATCGAAATCTTAATACATCCTTT 560
MK188360 Trissolcus japonicus      TCTATCTTTACCAGTTTTAGCAGGGGCAATCACTATAATTTTATCAGATCGAAATCTTAATACATCCTTT 560
                                   **********************************************************************

MK188349 Trissolcus japonicus      TTTAATCCAGCAGGA 575
MK188350 Trissolcus japonicus      TTTAATCCAGCAGGA 575
MK188360 Trissolcus japonicus      TTTAATCCAGCAGGA 575
                                   ***************
```

Fig. S1. Alignment of TJ234F/TJ460R primers against three *T. japonicus* sequences. *CO1* sequence alignment of three North American *T. japonicus* specimens used for primer design and location of primers TJ234F and TJ460R within the sequence.

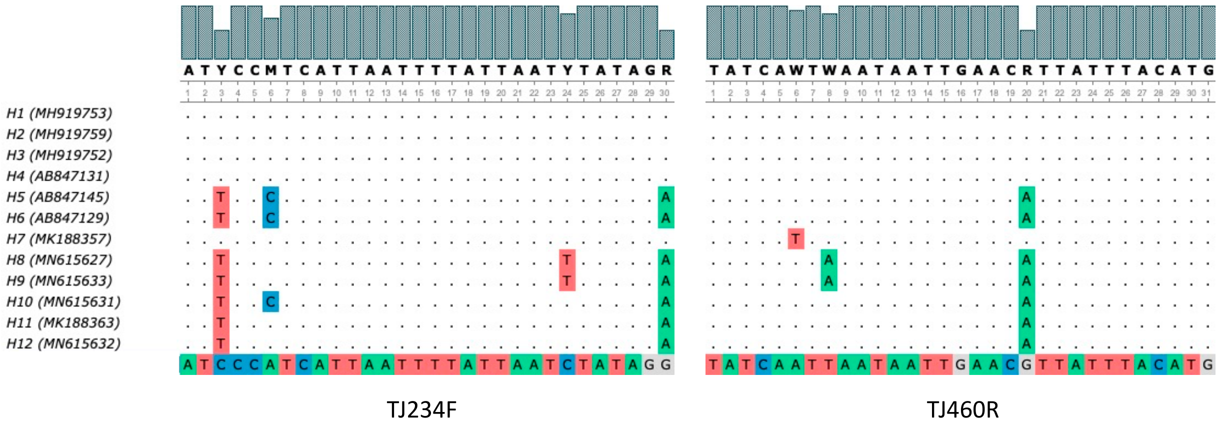

Fig. S2. Alignment between *T. japonicus CO1* haplotypes and TJ234F/TJ460R primer mismatches. Primers show total homology with ca. 71% of the sequences in the NCBI database, the ones belonging to

haplotypes H1-H4. These haplotypes include all the identified specimens from the US, Canada, Switzerland and Italy, plus variable proportions of Asian *T. japonicus* populations. Following the name of the haplotype, a representative NCBI accession number is specified. The haplotype names are based on Stahl et al. [55] and analysis in this study.

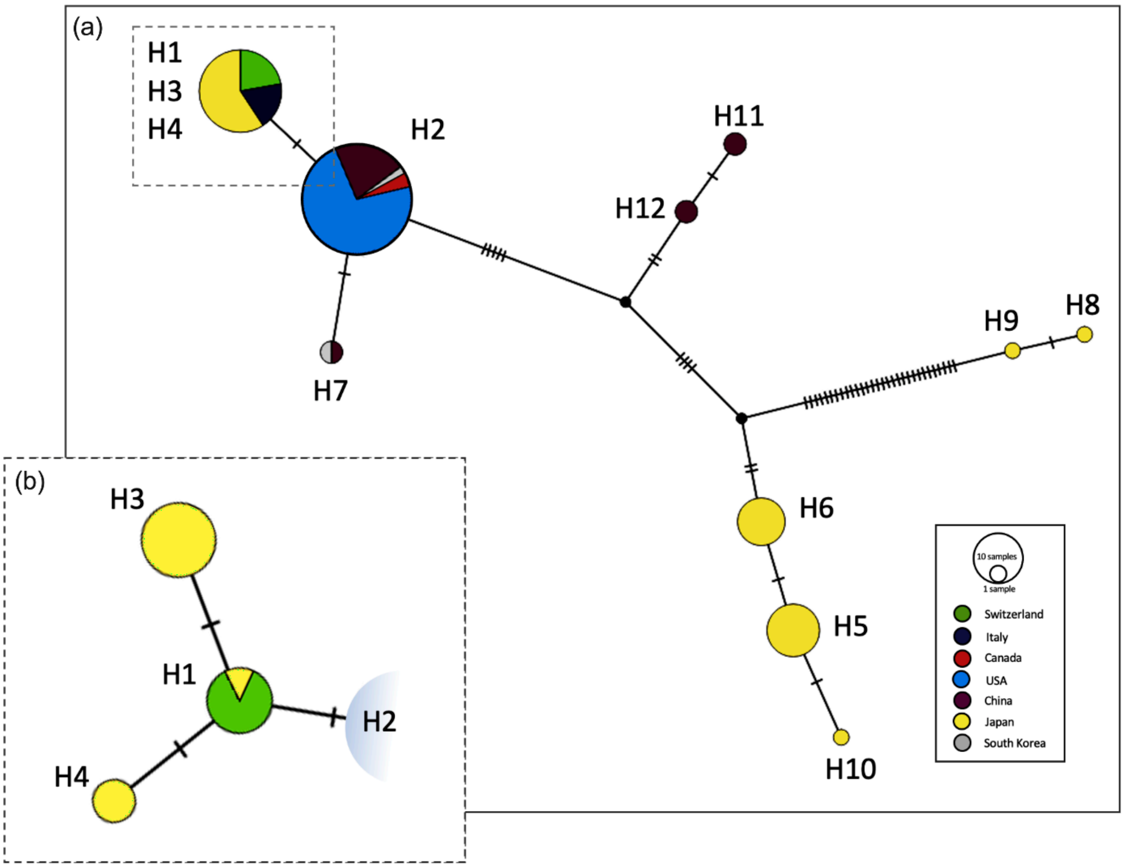

Fig. S3. Barcode haplotype network of the *T. japonicus CO1* sequences analyzed in this study. Each circle corresponds to one haplotype. Circle sizes are proportional to the number of sequences belonging to the haplotype, and each color distinguishes a different geographical origin. Black dots are nodes and hatch marks show the number of mutations between haplotypes. The phylogenetic relationships among haplotypes were established using statistical parsimony in TCS as implemented in PopART software [57]. The analyzed sequences had different lengths and were trimmed before the analysis. In (a), the haplotype network was constructed with 103 *T. japonicus CO1* sequences of 373 bp. The region of the analyzed *CO1* sequences did not allow distinguishing between the haplotypes H1, H3 and H4 described by Stahl et al. [55], who used longer *CO1* sequences for the analysis. In (b) the relationship between haplotypes H1, H3, and H4 described by Stahl et al. [55] is represented. In this case, the compared sequences are 423 bp long, and exclude the Italian and Japanese sequences whose short length did not allow their haplotype (H1, H3 or H4) to be elucidated.

Haplotype	Representative sequence	5	16	19	29	34	37	55	61	67	73	74	76	91	94	100	103	106	109	115	154	155	160	161	175
H1	MH919753	T	A	A	C	C	A	C	G	C	A	T	A	A	A	A	C	T	T	T	C	C	T	A	A
H2	MH919759	.	.	.	.	.	.	.	.	.	.	.	.	.	.	.	.	.	.	.	.	.	.	.	.
H3	MH919744	.	.	.	.	.	.	.	.	.	.	.	.	.	.	.	.	.	.	.	.	.	.	.	.
H4	AB847131	.	.	.	.	.	.	.	.	.	.	.	.	.	.	.	.	.	.	.	.	.	.	.	.
H5	AB847144	.	.	.	.	T	C	.	A	.	.	.	.	.	G	.	T	.	.	.	.	T	.	G	.
H6	AB847129	.	.	.	.	T	C	.	A	.	.	.	.	.	.	.	T	.	.	.	.	T	.	G	.
H7	MK188357	.	.	.	.	.	.	.	.	.	.	.	.	.	.	.	.	.	.	.	.	.	.	.	.
H8	MN615627	C	G	G	T	T	.	T	A	T	G	.	C	.	.	T	.	A	A	A	A	T	C	G	T
H9	MN615633	C	G	G	T	T	.	T	A	T	G	.	C	.	.	T	T	A	A	A	A	T	C	G	T
H10	MN615631	.	.	.	.	T	C	.	A	.	.	.	.	.	G	.	T	.	.	.	.	T	.	G	.
H11	MK188363	.	.	.	.	T	.	.	A	.	.	G	.	G	.	.	.	.	.	.	.	.	.	G	.
H12	MN615632	.	.	.	.	T	.	.	A	.	.	.	.	G	.	.	.	.	.	.	.	.	.	G	.

Haplotype	Representative sequence	178	187	196	217	233	235	247	278	281	284	286	289	290	310	313	319	329	343	349	355	358	364	367	370
H1	MH919753	T	A	T	T	A	T	G	A	T	T	A	T	C	C	G	C	T	T	T	C	T	T	A	A
H2	MH919759	.	.	.	.	.	.	.	.	.	.	.	.	.	A	.	.	.	.	.	.	.	.	.	.
H3	MH919744	.	.	.	.	.	.	.	.	.	.	.	.	.	.	.	.	.	.	.	.	.	.	.	.
H4	AB847131	.	.	.	.	.	.	.	.	.	.	.	.	.	.	.	.	.	.	.	.	.	.	.	.
H5	AB847144	.	.	.	.	.	.	A	.	.	.	.	C	.	A	A	.	.	.	.	T	.	C	.	.
H6	AB847129	.	.	.	.	.	.	A	.	.	.	.	C	.	A	A	.	.	.	.	T	.	C	.	.
H7	MK188357	.	.	.	.	T	.	.	.	.	.	.	.	.	A	.	.	.	.	.	.	.	.	.	.
H8	MN615627	A	G	C	C	.	A	A	.	C	C	T	C	T	A	A	T	C	C	C	.	.	C	G	T
H9	MN615633	A	G	C	C	.	A	A	.	C	C	T	C	T	A	A	T	C	C	C	.	.	C	G	T
H10	MN615631	.	.	.	.	.	.	A	.	.	.	.	C	.	A	A	.	.	.	.	T	C	C	.	.
H11	MK188363	.	.	.	.	.	.	A	G	.	.	.	.	.	A	A	.	.	.	.	.	.	.	.	.
H12	MN615632	.	.	.	.	.	.	A	G	.	.	.	.	.	A	A	.	.	.	.	.	.	.	.	.

Haplotype	Representative sequence	374	375	376	377	378	379	380	381	382	383	384	385	386	387	388	389	390	391	392	393	394	395	396	397
H1	MH919753	G	G	A	G	G	A	G	A	T	C	C	A	G	T	A	C	T	A	T	A	C	C	A	A
H2	MH919759	.	.	.	.	.	.	.	.	.	.	.	.	.	.	.	.	.	.	.	.	.	.	.	.
H3	MH919744	.	.	.	.	.	.	.	.	.	.	.	.	.	.	.	.	.	.	.	.	.	.	.	.
H4	AB847131	.	.	.	.	.	.	.	.	.	.	.	.	.	.	.	.	.	.	.	.	.	.	.	.
H5	AB847144	.	.	.	.	.	.	.	.	.	.	.	.	.	.	.	T	.	.	.	.	.	.	.	.
H6	AB847129	.	.	.	.	.	.	.	.	.	.	.	.	.	.	.	T	.	.	.	.	.	.	.	.
H7	MK188357	-	-	-	-	-	-	-	-	-	-	-	-	-	-	-	-	-	-	-	-	-	-	-	-
H8	MN615627	-	-	-	-	-	-	-	-	-	-	-	-	-	-	-	-	-	-	-	-	-	-	-	-
H9	MN615633	-	-	-	-	-	-	-	-	-	-	-	-	-	-	-	-	-	-	-	-	-	-	-	-
H10	MN615631	-	-	-	-	-	-	-	-	-	-	-	-	-	-	-	-	-	-	-	-	-	-	-	-
H11	MK188363	-	-	-	-	-	-	-	-	-	-	-	-	-	-	-	-	-	-	-	-	-	-	-	-
H12	MN615632	-	-	-	-	-	-	-	-	-	-	-	-	-	-	-	-	-	-	-	-	-	-	-	-

Haplotype	Representative sequence	398	399	400	401	402	403	404	405	406	407	408	409	410	411	412	413	414	415	416	417	418	419	420	421
H1	MH919753	C	A	T	T	T	A	T	T	T	T	G	A	T	T	T	T	T	T	G	G	T	C	A	C
H2	MH919759	.	.	.	.	.	.	.	.	.	.	.	.	.	.	.	.	.	.	.	.	.	.	.	.
H3	MH919744	.	.	.	.	.	.	.	.	.	.	.	.	.	.	.	.	.	.	.	.	A	.	.	.
H4	AB847131	.	.	.	.	.	.	.	.	.	.	.	.	.	.	.	.	.	.	.	.	.	.	.	T
H5	AB847144	.	.	.	.	.	.	.	.	.	.	.	.	.	.	.	.	.	.	.	.	.	.	.	.
H6	AB847129	.	.	.	.	.	.	.	.	.	.	.	.	.	.	.	.	.	.	.	.	.	.	.	.
H7	MK188357	-	-	-	-	-	-	-	-	-	-	-	-	-	-	-	-	-	-	-	-	-	-	-	-
H8	MN615627	-	-	-	-	-	-	-	-	-	-	-	-	-	-	-	-	-	-	-	-	-	-	-	-
H9	MN615633	-	-	-	-	-	-	-	-	-	-	-	-	-	-	-	-	-	-	-	-	-	-	-	-
H10	MN615631	-	-	-	-	-	-	-	-	-	-	-	-	-	-	-	-	-	-	-	-	-	-	-	-
H11	MK188363	-	-	-	-	-	-	-	-	-	-	-	-	-	-	-	-	-	-	-	-	-	-	-	-
H12	MN615632	-	-	-	-	-	-	-	-	-	-	-	-	-	-	-	-	-	-	-	-	-	-	-	-

Fig. S4. Position of haplotype markers in *T. japonicus CO1* sequence. For each haplotype, the NCBI accession number of a representative sequence is provided. Haplotype H1 was arbitrary selected as reference. Identical nucleotides are represented with dots. Differences between haplotypes H1-H6 were described by Stahl et al. [55] using *CO1* aligned sequences of 423 nucleotides. Sequences representing haplotypes H7-H12 are shorter (dashes in a grey background denote non-available *CO1* sequence regions) and can be differentiated from each other and from haplotypes H1-H6 by comparing a shorter region (373 bp).
